# Molecular Dynamics Simulation of Surface Properties in Styrene-Based Unsaturated Cardanol Sulfonate Surfactants

**DOI:** 10.1021/acsomega.5c13520

**Published:** 2026-04-17

**Authors:** Weiyang Liu, Congying Lu, Minjia Xia, Xin Zhao, Zuxi Zhang, Ruihong Huang, Fan Ling Xiao, Wei Ding

**Affiliations:** † Key Laboratory of Enhanced Oil Recovery, Ministry of Education, College of Petroleum Engineering, 117792 Northeast Petroleum University, Daqing, Heilongjiang 163318, China; ‡ 177520 Heilongjiang Key Laboratory of Oilfield Applied Chemistry and Technology of Daqing Normal University, Daqing, Heilongjiang 163712, China; § Exploration and Development Research Institute of Daqing Oilfield Recovery Research Room 2, Daqing, Heilongjiang 163712, China; ∥ Northeast Petroleum University, College of Chemistry and Chemical Engineering, Oil and Gas Chemical Technology Provincial Key Laboratory, Daqing, Heilongjiang 163318, China

## Abstract

The effects of blending ratio and molecular structure on the interfacial properties of styrene-based unsaturated cashew phenol sulfonate surfactants were systematically studied by molecular dynamics simulation. The research findings demonstrated that the single-benzene-ring system, characterized by its flexible molecular configuration, is capable of achieving vertical adsorption arrangement at the interface, forming high-density molecular packing layers, establishing stable hydrogen-bond networks, and significantly reducing surface tension. In the blending system, a higher proportion of the single-benzene-ring component facilitates the attainment of the optimal equilibrium between flexible chain segments and rigid structures, thereby enhancing the molecular arrangement order and solvation at the interface and further improving the overall performance. The synergistic effect of the quantity of hydrogen bonds and molecular spatial order is a crucial factor in regulating interfacial stability. This study offers important theoretical foundations and methodological support for the molecular design and performance optimization of biobased surfactants.

## Graphical abstract

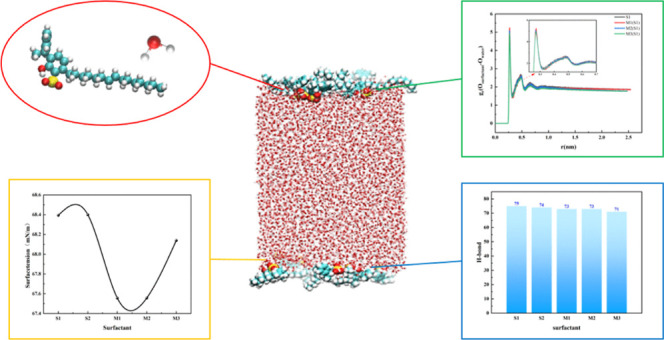

## 1 Introduction

Compared to petroleum-derived surfactants, biobased surfactants offer greater economic and environmental benefits, demonstrating significant potential in supporting global “dual carbon” objectives and advancing the principles of greenchemical engineering, along with a broad market for applications.^1,2^ Biobased surfactants derived from structural modifications of cardanol exhibit superior performance, excellent environmental compatibility, and access to abundant raw material resources, positioning them as widely recognized green and sustainableas widely recognized green and sustainable alternatives.^3^ Cashew nut shell liquid (CNSL), a byproduct of the cashew processing industry, is one of the primary sources of natural phenols and is extracted from the shells of cashew nuts. Originating in Brazil, the cashew tree is now extensively cultivated in tropical regions, including India, Mozambique, Madagascar, Tanzania, and the Philippines. The shells contain approximately 30–35% CNSL, which constitutes about 67% of the total nut weight.^4^ The main constituents of CNSL are phenolic compounds such as anacardic acid, cardanol, and cardols.^5^ Owing to their inherent phenolic characteristics and variable degrees of unsaturation in the aliphatic side chains attached to the benzene ring, these components find diverse applications across industries, including food, materials, industrial processes, and pharmaceuticals.^6,7^

The molecular structure of cardanol features both hydrophilic phenolic hydroxyl groups and hydrophobic long-chain alkyl moieties. This amphiphilic architecture, together with its natural abundance and low toxicity, renders cardanol a highly favorable biobased building block for chemical synthesis.^8^ The phenolic hydroxyl group is chemically versatile, enabling a wide range of transformations such as esterification,^9^ etherification,^10,11^ and nucleophilic substitution reactions.^12,13^ Concurrently, the hydrophobic alkyl chain not only enhances the lipophilicity of derived surfactants but also participates in various functionalization reactions, including epoxidation,^9,10^ hydrogenation,^14,15^ carbonation,^16^ thiol–ene addition,^17,18^ and Diels–Alder cycloadditions,^19^ thereby allowing precise modulation of key properties such as surface activity, emulsification stability, and biocompatibility.

In the development of cardanol-based modified surfactants, derivatives of various ionic types have exhibited diverse application potentials. Cationic cardanol surfactants, obtained by introducing quaternary ammonium groups, leverage their positive charge to demonstrate exceptional performance in antibacterial materials. For example, Zhao et al.^20^ synthesized a series of cardanol-derived quaternary ammonium salts (HCQAS), which exhibit excellent surface activity—evidenced by low critical micelle concentration and low surface tension—along with favorable wetting properties and inherent antibacterial efficacy. These characteristics render HCQAS suitable for use in antibacterial coatings and detergents, thereby offering new avenues for the functional application of cardanol-based surfactants. Anionic cardanol surfactants, benefiting from the strong hydrophilicity of sulfonic or carboxylic acid groups, are widely employed in industrial cleaning applications. Chen et al.^21^ developed a novel anionic–nonionic surfactant (CPES) based on cardanol polyoxyethylene ether, using maleic anhydride and sodium bisulfite as coreactants. This surfactant exhibits a low critical micelle concentration (CMC) of 1.61 × 10^–4^ mol/L and achieves a surface tension of 34.54 mN/m, while maintaining good stability across varying pH and saline conditions. When formulated into aluminum cleaning agents, CPES demonstrates noncorrosive behavior, environmental compatibility, and superior cleaning efficiency, presenting a viable alternative to conventional petroleum-derived surfactants. Nonionic cardanol surfactants, typically functionalized with polyoxyethylene chains or similar moieties, exhibit strong emulsifying capacity and biocompatibility, attracting significant interest in pesticide adjuvant applications. Denis et al.^22^ incorporated phosphorylated cardanol as a reactive diluent into short-oil alkyd resins, effectively reducing system vis- cosity—thereby decreasing solvent demand by approximately 9 wt %—while simultaneously imparting enhanced flame retardancy (reducing peak heat release rate by 41% compared to traditional resins) without compromising essential coating properties such as flexibility. This work provides valuable insights into the utilization of nonionic cardanol derivatives in environmentally friendly coating systems. Collectively, these studies underscore the feasibility and versatility of cardanol-based surfactants across different ionic configurations.

Building upon the molecular structure of cardanol, this study incorporates sulfonate group to enhance its hydrophilicity and surface activity. The introduction of styryl groups is designed to further modulate molecular rigidity, conformation of the hydrophobic chain, and interfacial packing behavior, thereby improving surfactant performance. The resulting styryl-functionalized unsaturated cardanol sulfonate surfactant integrates the natural hydrophobicity of cardanol, the strong hydrophilicity of sulfonate groups, and the structural rigidity imparted by styryl moieties (Figure 1). This synergistic combination of functional groups endows the surfactant with distinct advantages in reducing surface tension, stabilizing emulsions, and exhibiting resistance to elevated temperatures and high salinity. However, the macroscopic properties of surfactants are fundamentally governed by their micro- scopic interfacial behavior, including interfacial characteristics, aggregation morphologies, and intermolecular interactions. Relying solely on experimental approaches is insufficient to elucidate the underlying molecular mechanisms of performance modulation at the atomic level.

**Figure 1:**
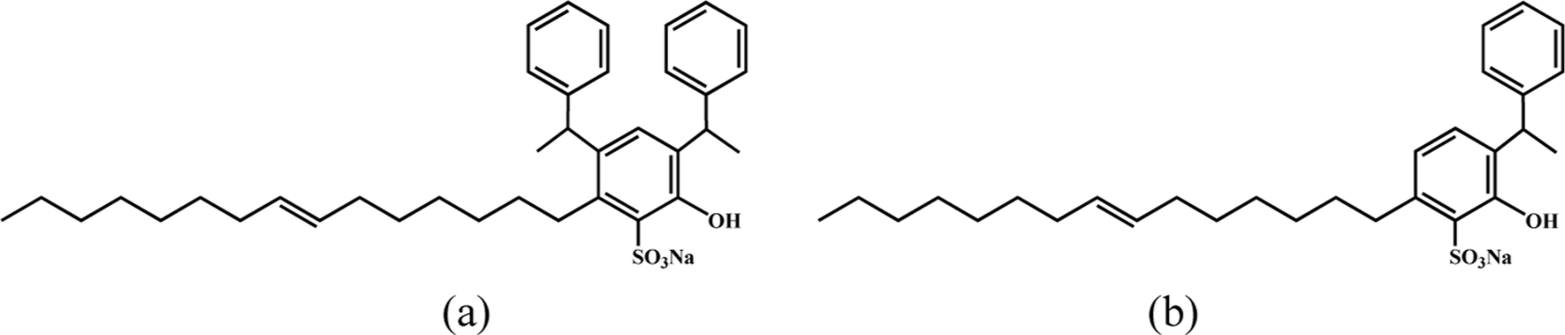
Schematic diagram of surfactant structure: (a) D-SUCS; (b) M-SUCS.

Molecular dynamics simulation, as a powerful computational tool for probing molecular behavior at the microscopic scale, overcomes the spatiotemporal limitations of experimental observation and enables accurate modeling of the dynamic evolution of surfactant molecules in solution and at interfaces.^23^ By constructing a molecular model of styrene-based unsaturated cardanol sulfonate and establishing a well-defined simulation system—such as aqueous solution or oil/water interface—with appropriate force field parameters,^24^ the diffusion, self-aggregation, and molecular arrangement behaviors under varying concentrations, temperatures, and ionic strengths can be systematically tracked. This provides essential data support and a theoretical foundation for elucidating the mechanisms of surfactant interfacial interactions from a molecular-level perspective.^25^ In recent years, the application of molecular dynamics simulations to investigate surfactant interfacial aggregation has grown significantly. For instance, Lu et al.^3^ developed a surfactant model and employed GROMACS software combined with the GAFF force field to simulate the coadsorption behavior of alcohols with different alkyl chain lengths and dodecylamine at the gas/water interface. Through analysis of density distribution profiles and radial distribution functions, they revealed that the synergistic interaction between the two components facilitates the formation of a dense hydrophobic film. Hou et al.^26^ investigated the adsorption behavior of various surfactants on carbonate rock surfaces using both experimental methods and molecular dynamics simulations. Their results showed that cationic surfactants exhibit higher adsorption capacity than anionic counterparts, that elevated temperature suppresses adsorption. In contrast, high salinity enhances it, and that the addition of 1000 kg/m^3^ O_2_ nanoparticles reduces surfactant adsorption. Jiao et al.^27^ examined the mixed system of the short-chain fluorocarbon surfactant PFHXA and the anionic surfactant SDS at the gas/water interface via molecular dynamics simulation. They found that the two surfactants synergistically form a compact monolayer and that incorporation of the fluorocarbon component reduces the tilt angle of SDS molecules at the interface. Collectively, these studies demonstrate that molecular dynamics simulation is capable of uncovering the interfacial behavior and structure–property relationships of surfactants at the atomic level, thereby establishing a robust methodological and theoretical framework for the investigation of styrene-based unsaturated cardanol sulfonate surfactants.

This study employs the molecular dynamics simulation method to investigate the interfacial behavior of styrene-based unsaturated cardanol sulfonate surfactants. Molecular models with varying blending ratios were constructed, and a gas/water interface simulation system was established using appropriate force field parameters to examine their dynamic behavior at the gas–liquid interface. By tracking the diffusion, self-aggregation, and molecular arrangement processes across different blending ratios, and integrating analyses of density distribution, radial distribution functions, and molecular orientation, the influence of blending ratios on interfacial aggregation morphology and packing density was systematically elucidated. Furthermore, the underlying microscopic mechanism of synergistic interactions was clarified. The research aims to reveal the structure–property relationship of the blended surfactant system at the molecular level, thereby providing a theoretical basis for optimizing formulation strategies and enhancing surface performance.

## 2 Calculation Method

### 2.1 Force Field

This study employed the General AMBER Force Field (GAFF)^28,29^ for molecular dynamics simulations. GAFF’s universal parameters for organic and bioorganic systems precisely fit the benzene ring, alkyl chain, and sulfonate group of styrene-based unsaturated cardanol sulfonate, reliably describing its bonding (bond stretching, angle bending, dihedral rotation) and nonbonding interactions (van der Waals forces, electrostatic interactions). The electrostatic potential charges fitted based on the RESP method, with the introduction of a charge restraint term to suppress the fluctuation of buried atom charges, ensure the accuracy of intermolecular electrostatic interaction calculations while enhancing the stability of charge changes with conformation, facilitating the simulation of conformational changes in molecules during dynamic processes. Combined with the SPCE water model,^30^ the two work in synergy to accurately depict the polar environment at the gas/water interface: SPCE water molecules accurately reflect the hydrogen bonding between water and sulfonate group and the hydrophobic effect of alkyl chains, and are compatible with GAFF parameters, avoiding parameter mismatch issues in cross-force field simulations. This combination maintains the stability of the system during long-term simulations, clearly presenting the adsorption orientation, aggregation behavior, and conformational adjustments of molecules at the interface, providing a highly reliable simulation basis for revealing their interfacial interaction mechanisms.

### 2.2 Simulation Details

Packmol^31^ was used to construct the vacuum surfactant–water model (Figure 2). There were a total of 6,672 water molecules in the central region of the model, with a total of 12 surfactant molecules (6 on each side) symmetrically arranged at the interfaces of the aqueous phase, ensuring proper orientation of the hydrophilic headgroups toward the water and the hydrophobic carbon chains extending toward the air, thereby promoting stable monolayer formation at both sides of the water slab, and there was a 0.1 nm overlap between adjacent molecules. Additionally, a defined vacuum region was maintained on both sides of the surfactant. The cross-sectional area of the simulation box was 5.0 × 5.0 nm^2^, and its length was extended to 24.0 nm. Considering that the surfactant was anionic, 12 sodium ions were introduced into the system as counterions to maintain electrical neutrality and effectively avoid electrostatic interference, ensuring the stability and accuracy of subsequent simulations. The modeling details are shown in Figure 2. First, the steepest descent method was used for energy minimization; then, 1 ns of isothermal–isobaric (*NPT*) simulation (298.15 K, 101.325 kPa) and 10 ns of canonical ensemble (*NVT*) simulation were performed, with a step size of 2 fs. The last 5 ns of the trajectory were used for data analysis. First, 1 ns of *NPT* simulation (298.15 K, 101.325 kPa) was carried out using the Berendsen^32^ pressure coupling method, with a relaxation time of 1 ps, to bring the system to equilibrium at the target pressure. Subsequently, 10 ns of NVT simulation (298.15 K) was conducted using the V-rescale^33^ thermostat to control the temperature. Molecular bond lengths were effectively constrained using the LINCS algorithm.^34,35^ Short-range interactions were calculated using the Lennard-Jones potential function with a cutoff radius of 1.2 nm, and long-range electrostatic interactions were treated using the particle mesh Ewald (PME) summation method.^36^ The water model was selected as SPC/E.^30^ All molecular dynamics simulations were performed using Gromacs (2020)^37^ software. All key modeling settings are shown in Table 1.

**Figure 2:**
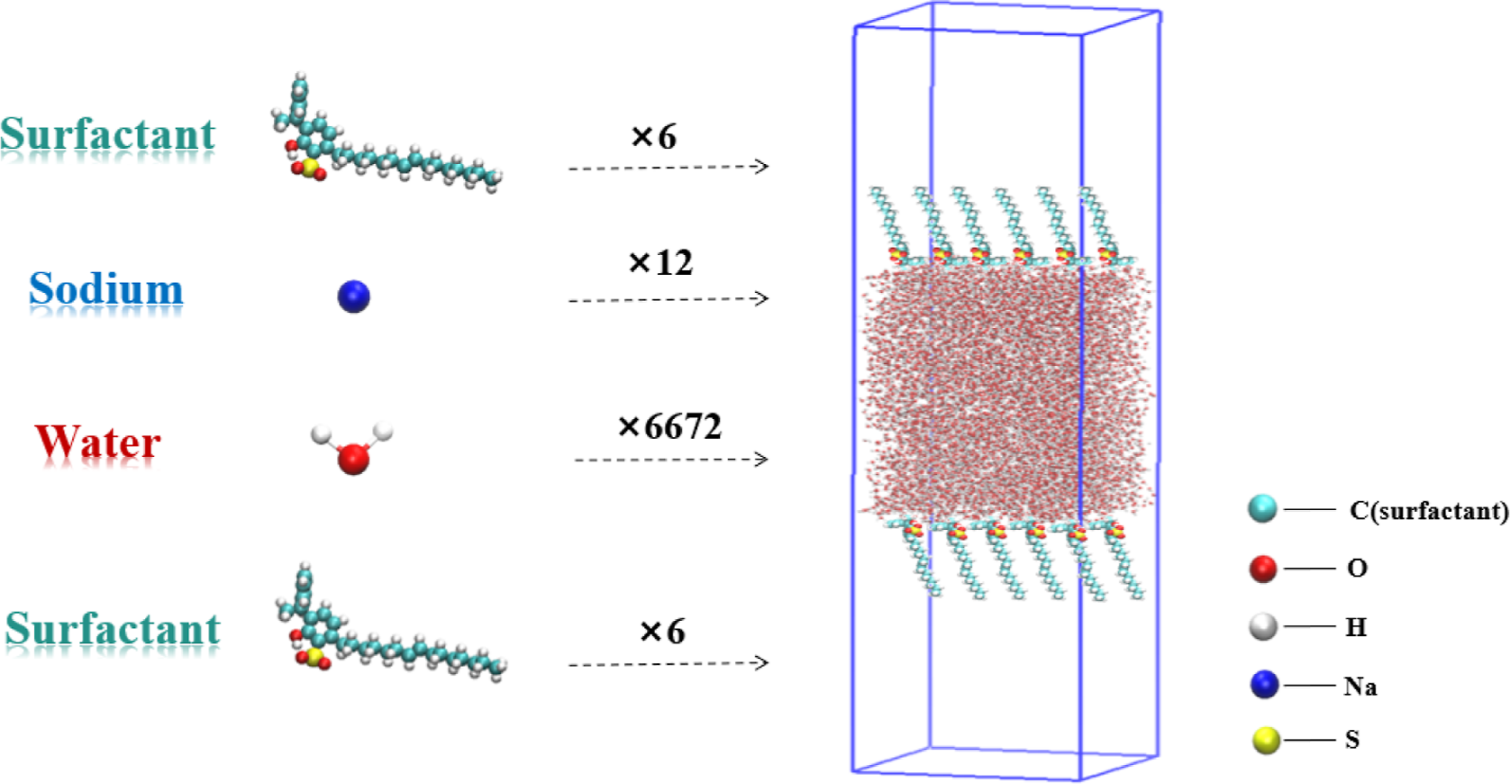
Schematic diagram of model construction.

**Table 1:** All Key Modeling Settings

category	parameter	value/description
system type	surfactant monolayer	at air/water interface
surfactant models	monophenyl (flexible),bis-phenyl (rigid)	M1: 9:3 M2: 3:2 M3: 3:9
force field	All-atom	GAFF
water model	TIP3P/SPC/E	SPC/E
box dimensions	initial size (*x* × *y* × *z*)	e.g., 5.0 × 5.0 × 24.0 nm3
number of molecules	surfactants	12 (all system)
water molecules	6672 (ensuring sufficient hydration)	
sodion	12(to maintain electrical neutrality and effectively avoid electrostatic interference)	
temperature	thermostat	298.15 K (*NPT* or *NVT* ensemble)
pressure	barostat	1 atm (for NPT simulations)
electrostatics	treatment	Particle Mesh Ewald (PME)
van der Waals	cutoff	1.0–1.2 nm with switching function
bond constraints	hydrogen atoms	LINCS algorithm
time step	integration step	2 fs
equilibration	duration	1–5 ns
production run	duration	5–10 ns
trajectory sampling	frequency	every 10 ps
software package	MD engine	GROMACS 2020

## 3 Results and Discussion

Building upon the previously established molecular dynamics simulation system for the styrene-based unsaturated cardanol sulfonate (SUCS) gas/water interface, this study systematically investigates the effects of blending ratio (moleculus ratio: 9:3, 3:2, 3:9) and structural differences between monophenyl (M-SUCS) and diphenyl (D-SUCS) surfactants on interfacial behavior. The analysis is carried out from three perspectives: surface properties, adsorption morphology, and intermolecular inter- actions. Surface tension data are employed to examine the regulatory influence of blending ratios on surface activity. Density distribution profiles reveal the differences in aggregation density and molecular orientation of M-SUCS and D-SUCS at the interface. Furthermore, the interaction mechanism between sulfonate group and water molecules is elucidated by integrating radial distribution functions with hydrogen bond population analysis. The study aims to uncover the synergistic regulation mechanism of blending ratio and SUCS molecular structure on interfacial behavior at the molecular level, thereby providing a theoretical foundation for understanding the gas/water interfacial characteristics of M-SUCS and D-SUCS and for optimizing their formulation strategies.

First, to evaluate the structural stability and dynamic equilibrium of the simulated systems, the root mean square deviation (RMSD) of all surfactant molecules relative to their initial configurations was calculated throughout the 10 ns production run. RMSD serves as a critical metric for assessing conformational convergence, indicating whether the system has relaxed into a stable thermodynamic state.

As shown in Figure 3, the RMSD plots for all systems (S1, S2, M1, M2, and M3) exhibit a characteristic two-stage evolution. During the initial 1000 ps, the RMSD values increase rapidly, reflecting the initial structural relaxation and conformational rearrangement of the surfactant molecules as they adapt to the solvent environment. Following this initial adjustment, the RMSD curves for all systems reach a plateau and fluctuate around stable average values (approximately 1.5–3.5 nm) for the remainder of the simulation time. The absence of systematic drift in the RMSD after 1 ns confirms that all systems have achieved dynamic equilibrium. Consequently, the subsequent analyses, including radial distribution functions and hydrogen bonding dynamics, are based on this equilibrated trajectory, ensuring the statistical significance and physical relevance of the reported results.

**Figure 3:**
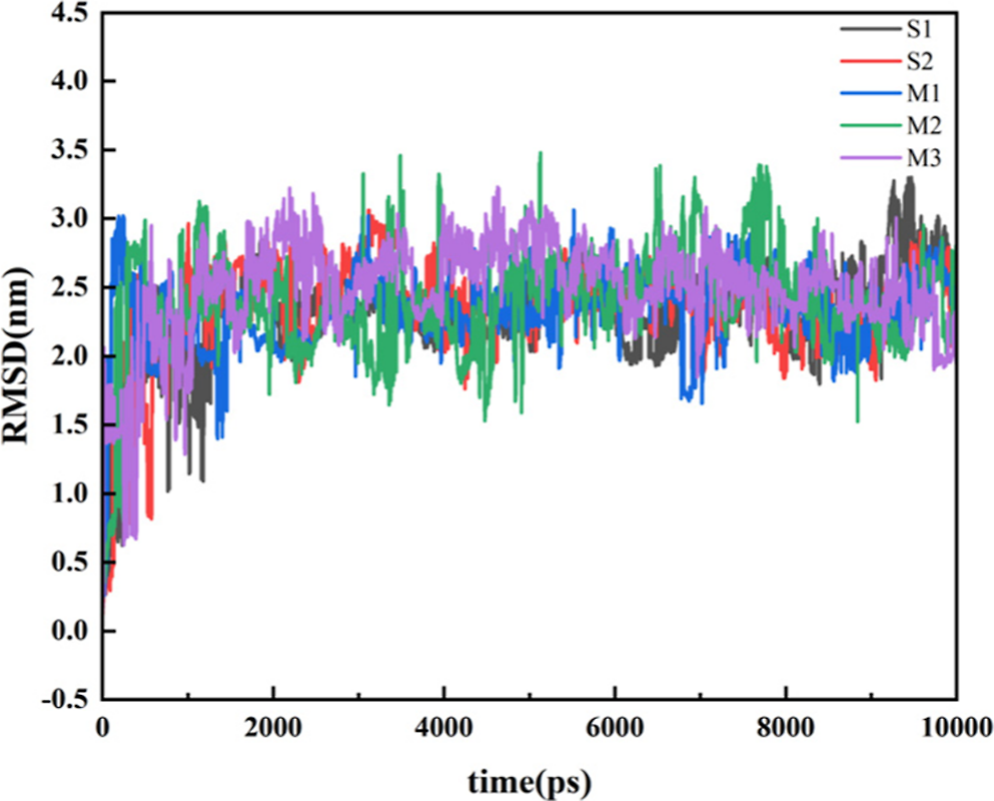
RMSD curves of each system.

### 3.1 Surface Properties

#### 3.1.1 Density Distribution

Density distribution reveals the degree of molecular aggregation at the interface, while surface tension reflects the ability of surfactant molecules to reduce interfacial energy and form stable interfacial films. By analyzing the differences in density distribution and surface tension between single-component systems (S1/S2) and blended systems (M1/M2/M3), this study systematically elucidates the evolution patterns and underlying mechanisms of surface properties across two critical dimensions: molecular structure (monophenyl vs diphenyl) and blending ratio (monophenyl/diphenyl proportion).

Figure 4 presents the density distribution curves of different systems at the gas/water interface, where the horizontal axis (*r*) denotes the normal distance from the interface and the vertical axis represents the normalized density. Figure 5 provides an enlarged view of the density distribution for each individual system.

**Figure 4:**
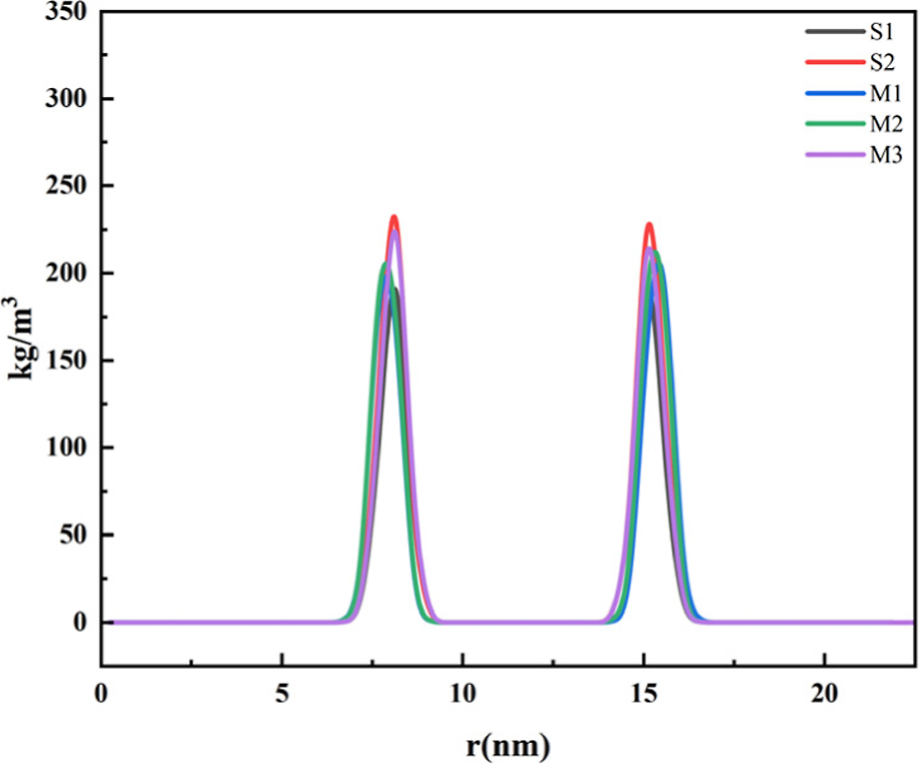
Density distribution curves of each system.

**Figure 5:**
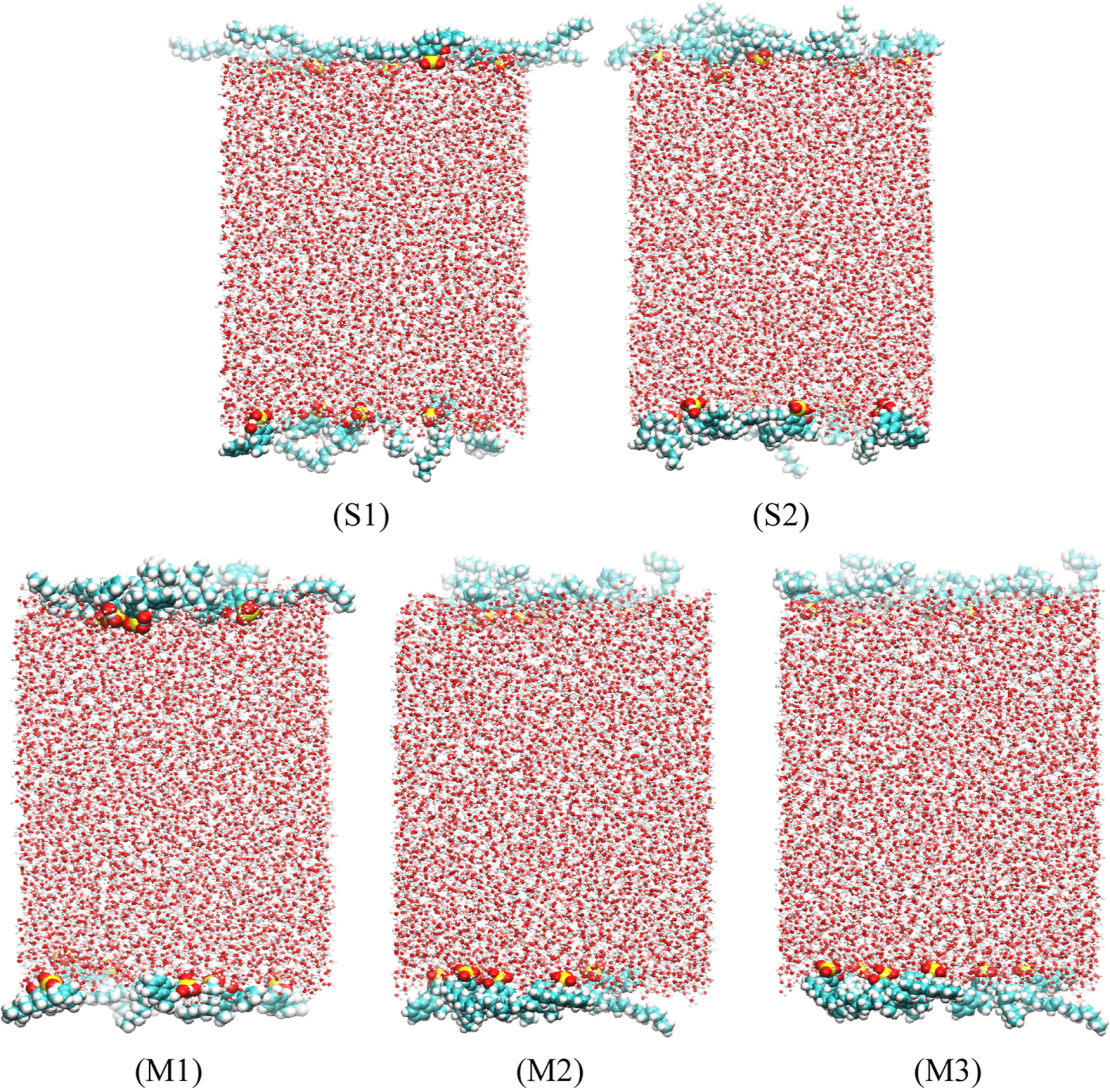
Equilibrium configuration snapshot.

S1 (monophenyl): The structural rigidity is the primary factor governing the differences in density distribution within a single system. The density distribution curve of S1 exhibits a high and narrow peak, with a maximum value of approximately 300 kg/m^3^ and a full width at half-maximum of about 2 nm. The peak positions are localized at the gas/water interface, corresponding to a bilayer arrangement. The flexible alkyl chain and sulfonate group (−SO_3_^–^) in the single phenyl structure enable close molecular packing through conformational adjustments: the hydrophobic benzene ring and alkyl chain orient upward, forming a compact hydrophobic layer, while the hydrophilic sulfonate group extends downward into the aqueous phase, resulting in a “vertical” adsorption configuration. This high-density, narrow distribution indicates a high interfacial adsorption density, tight molecular aggregation, and minimal intermolecular voids—features that contribute to the formation of a stable interfacial film.

S2 (Diphenyl): the density distribution curve displays a lower and broader peak, with a maximum value of approximately 220 kg/m^3^ and a fwhm of about 4 nm, with peak positions similarly located at the interface. The increased rigidity of the double phenyl structure—featuring two benzene rings linked by an ethylene group—restricts conformational flexibility, thereby impeding efficient molecular packing. This rigidity enhances steric hindrance among the hydrophobic moieties (dual benzene rings and alkyl chain), necessitating greater intermolecular spacing and leading to a “dispersed” adsorption pattern. Furthermore, the restricted rotational freedom limits optimal orientation at the interface, resulting in a “tilted” or “lying” molecular arrangement. These effects manifest as a reduced peak height and an expanded peak width in the density distribution. Compared to S1, the peak value for S2 decreases by approximately 26.7%, while the peak width increases by 100%, indicating lower interfacial adsorption density, disordered molecular aggregation, and significantly enlarged intermolecular voids.

A synergistic effect of compositional regulation on density distribution is observed in the blended system. The density distribution exhibits a clear dependence on the mixing ratio, primarily governed by the conformational complementarity and spatial competition between the single phenyl (flexible) and double phenyl (rigid) components.

M1 (1-B-SO_3_: 2-B-SO_3_ = 9:3, high single phenyl proportion): the density distribution curve displays a high-density, narrow peak with a maximum value of approximately 290 kg/m^3^ and a full width at half-maximum of about 2.5 nm. The dominance of single phenyl molecules leads to strong interfacial adsorption, while their flexible alkyl chains effectively fill the voids created by the double phenyl groups, promoting dense molecular packing. Concurrently, the limited number of rigid double phenyl structures provides structural support that stabilizes the vertical orientation of the single phenyl molecules, reducing conformational fluctuations and enhancing overall packing efficiency. Compared to S1, the peak height decreases only by 3.3%, whereas the peak width increases by 25%, indicating that the high single phenyl composition maintains a high adsorption density, with intermolecular compactness further optimized through rigid structural reinforcement.

M2 (1-B-SO_3_: 2-B-SO_3_ = 3:2, medium proportion): the density distribution is characterized by a medium-density, broad peak, with a maximum value of approximately 200 kg/m^3^ and a fwhm of about 3.5 nm. At this balanced ratio, the numbers of flexible and rigid molecules are comparable, leading to competitive interactions at the interface. However, the conformational flexibility of the single phenyl units cannot fully accommodate the constraints imposed by the rigid double phenyl moieties, resulting in molecular mismatches, increased disorder, and the formation of intermolecular voids. This imbalance reduces the adsorption density—reflected in a 33.3% decrease in peak height relative to S1—and expands the peak width by 75%. This “medium-density broad peak” profile indicates suboptimal molecular aggregation and a significant reduction in the structural integrity of the interfacial film.

M3 (1-B-SO_3_: 2-B-SO_3_ = 3:9, high double phenyl proportion): the density distribution shows a low-density, broad peak, with a maximum value of approximately 180 kg/m^3^ and a fwhm of about 4.5 nm. The interfacial behavior is dominated by the double phenyl component, whose rigidity enforces a dispersed adsorption mode. The spatial hindrance introduced by these rigid structures prevents the flexible single phenyl molecules from achieving efficient packing, further decreasing the adsorption density (a 40% reduction compared to S1) and increasing the peak width by 125%. This “low-density broad peak” distribution demonstrates that, under high double phenyl loading, molecular compactness is severely compromised, resulting in the largest intermolecular voids and the weakest potential for forming a cohesive interfacial film.

#### 3.1.2 Surface Tension

Figure 6 presents the surface tension data for different systems, with the horizontal axis representing the composition ratios of the blended systems and the vertical axis denoting surface tension. Surface tension is a key indicator of surfactant performance, reflecting the ability of molecules to reduce interfacial energy and form stable interfacial films,^38,39^ its magnitude directly correlates with molecular surface activity. To validate the accuracy of our simulation setup, we first established a baseline by simulating a pure SPC/E water slab under identical conditions (box size, PME parameters, and cutoff distances) as the surfactant systems. The resulting surface tension was calculated to be 72.5 ± 2.0 mN/m. This value is in close agreement with the experimental value of 72.8 mN/m at 25 °C and aligns well with previously reported simulations using the SPC/E model. This validation confirms the reliability of our computational framework, ensuring that the subsequent reduction in surface tension observed upon surfactant adsorption is a genuine effect of the molecular interactions at the interface. A clear causal relationship exists between the surface tension values across systems and their corresponding density distributions—the height and width of the density peaks jointly govern the extent of surface tension reduction.Utilizing the Kirkwood–Buff (KB) method,^40^ this research computes surface tension and implements a three-step analytical framework via a comparative study of different surfactant formulations. Initially, the surface activity baseline of single components is anchored. Subsequently, synergistic effects are verified to screen for the optimal composition. Ultimately, microscopic mechanisms—including interfacial adsorption configurations and intermolecular forces—are integrated to elucidate the governing relationship between composition, surface tension, and surface activity.

**Figure 6:**
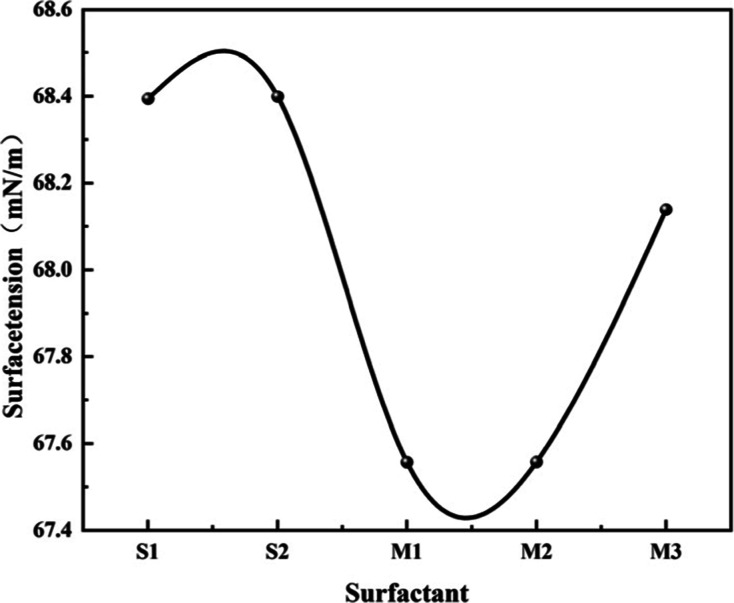
Surface tension curve graph.

S1 (monophenyl): the surface tension is 68.4 mN/m. The high-density, narrow-peak density distribution enables the formation of a densely packed adsorption layer at the interface. The hydrophobic moieties (benzene ring and alkyl chain) orient upward, effectively minimizing the interfacial area exposed to water and reducing surface energy, while the hydrophilic sulfonate group extend into the aqueous phase and form hydrogen bonds, thereby significantly lowering the surface tension. This value corresponds directly to the “high-density sharp peak” observed in the density distribution, indicating that the flexible molecular architecture achieves optimal surface activity through efficient close packing.

S2 (diphenyl): the surface tension is 68.5 mN/m. The low-density, broad-peak density distribution results in a less compact interfacial film. The dispersed arrangement of hydrophobic groups reduces their effectiveness in shielding the aqueous phase, while the reduced contact area between hydrophilic groups and water limits hydrogen bonding, leading to a marginally higher surface tension compared to S1. This surface tension value correlates with the “low-density wide peak” in the density distribution, demonstrating that molecular rigidity impairs surface activity due to a “dispersed adsorption” configuration.

M1: surface tension is 66.9 mN/m, the lowest among all systems. The high-density, narrow-peak distribution facilitates the formation of the most compact interfacial film, maximizing both the upward aggregation efficiency of hydrophobic groups and the solvation degree of hydrophilic groups, thus minimizing surface energy. Compared to S1, the surface tension decreases by 1.5 mN/m, indicating that the high monophenyl composition enhances surface activity through a synergistic effect between flexible and rigid components.

M2: surface tension is 67.6 mN/m. The medium-density, broad-peak distribution leads to a less dense interfacial layer, accompanied by reduced efficiency in hydrophobic group orientation and weakened hydrophilic solvation. As a result, the surface tension increases by 0.7 mN/m relative to M1, yet remains lower than that of S2. This value aligns with the “medium-density wide peak” profile, reflecting intermediate surface activity under balanced compositional conditions.

M3: surface tension is 68.1 mN/m. The low-density, broad-peak distribution results in the least compact interfacial film, with minimal hydrophobic exposure efficiency and the weakest hydrophilic solvation. Consequently, the surface tension increases by 1.2 mN/m compared to M1 and approaches that of S2. This observation corresponds to the “low-density wide peak” in the density distribution, indicating that excessive diphenyl content compromises surface activity due to dominant molecular rigidity.

Styrene-based unsaturated cardanol sulfonate surfactants exhibit distinct interfacial behaviors at the gas/water interface depending on their molecular architecture and blending ratios. S1, with its flexible structure, forms a highly dense and compact molecular arrangement, leading to low surface tension. In contrast, S2, constrained by structural rigidity, displays a broader density distribution and slightly higher surface tension due to less efficient packing. Among the blended systems, M1 achieves the densest interfacial aggregation and the lowest surface tension through synergistic interactions between flexible and rigid components. M2 exhibits disordered molecular organization arising from competitive adsorption, while M3 shows a low-density, dispersed configuration dominated by rigid diphenyl structures.

This consistent relationship between density distribution characteristics and surface tension reveals the underlying microscopic mechanism—enhanced interfacial condensation and tighter molecular packing result in superior surface activity. These findings demonstrate that formulations with a high proportion of monophenyl (monostyrene) units are optimal for achieving maximum surface tension reduction, providing a clear strategy for tuning the performance of such surfactant systems.

### 3.2 Adsorption Forms

#### 3.2.1 Tilt Angle

The tilt angle in our study is defined as the angle between the vector formed by two key carbon atoms along the cardanol alkyl chain and the *z*-axis, which typically represents the direction of the surface normal or the primary symmetry axis in the system. The reference vector is constructed by connecting the carbon atom at the terminal end of the long alkyl chain (e.g., the methyl group, C terminal) and the carbon atom directly bonded to the benzene ring (i.e., the first carbon in the alkyl chain, C benzylic); this C terminal–C benzylic vector effectively represents the overall orientation of the alkyl chain. The tilt angle is then calculated as the angle between this vector and the *z*-axis, as illustrated in the Figure 7. This choice of atoms is physically meaningful because the conformation and orientation of the alkyl chain are primarily determined by the spatial relationship between its anchored point (C benzylic) and its distal end (C terminal), allowing the vector to capture the average extension direction of the chain while minimizing the influence of local bond fluctuations, and thereby enabling consistent and reproducible measurements across different molecular configurations and simulation frames. The tilt angle is directly correlated with the exposure efficiency of hydrophobic groups, the solvation degree of hydrophilic groups, and the compactness of the interfacial film. A smaller tilt angle corresponds to a more “vertical” molecular orientation at the interface, which enhances the upward aggregation efficiency of hydrophobic moieties, promotes complete downward extension and solvation of hydrophilic groups, and results in a denser and more cohesive interfacial film. Conversely, a larger tilt angle indicates a more “tilted” or “lying” molecular configuration, leading to dispersed exposure of hydrophobic groups, reduced contact area between hydrophilic groups and the aqueous phase, and a looser, less organized interfacial structure. Significant variations in tilt angles are observed across different systems, primarily governed by molecular rigidity and blending ratio (Figure 8).

**Figure 7:**
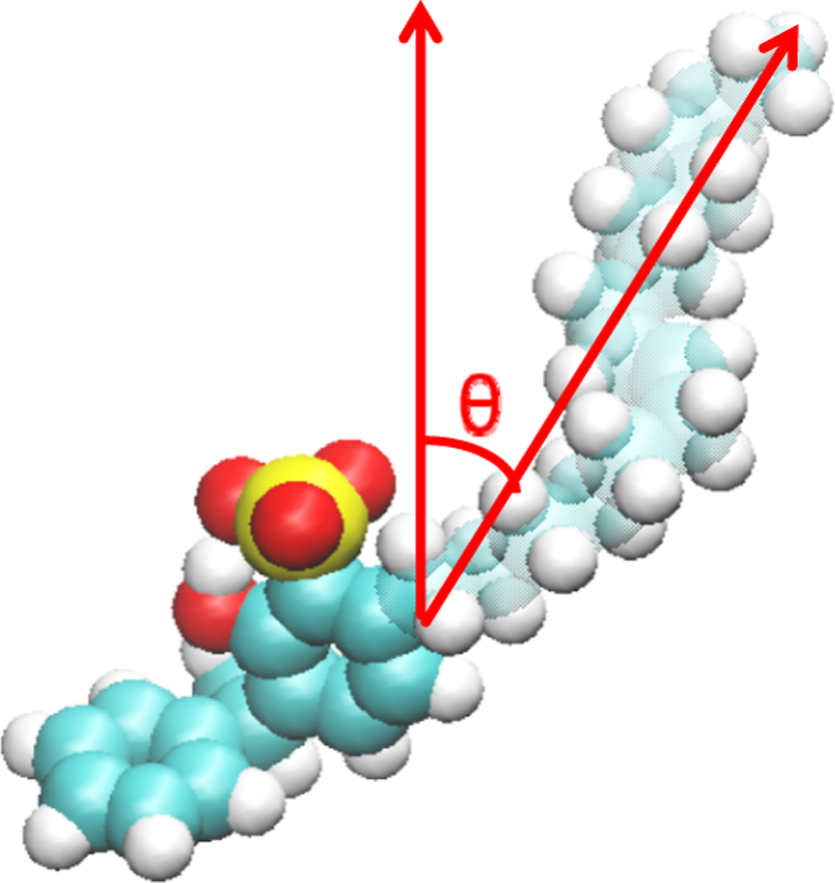
Diagram of the tilt angle.

**Figure 8:**
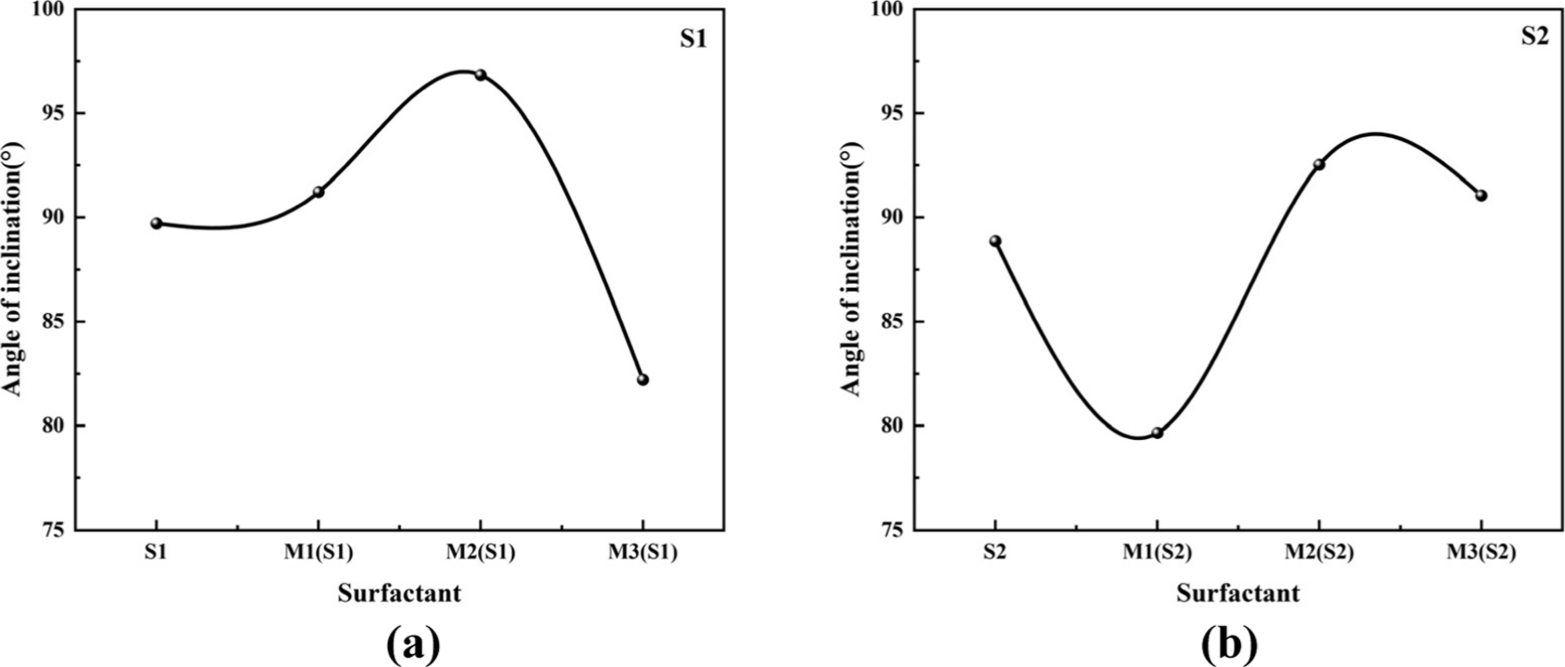
Tilt angle curves of surface activity for each system: (a) is the tilt angle curve of S1 in the surface activity of each system; (b) is the tilt angle curve of S2 in the surface activity of each system.

S1: the tilt angle is approximately 80°. The flexible alkyl chain of the single phenyl structure enables vertical adsorption through conformational adjustment, facilitated by the sulfonate group (−SO_3_^–^). In this configuration, the hydrophobic benzene ring and alkyl chain orient upward to form a “dense hydrophobic layer,” while the hydrophilic sulfonate group extends downward into the aqueous phase, minimizing the angle between the molecular axis and the interfacial normal. This near-vertical adsorption orientation maximizes both the exposure efficiency of hydrophobic moieties and the solvation degree of hydrophilic groups, thereby promoting the formation of a dense and cohesive interfacial film.

S2: the tilt angle is approximately 90°. The rigidity of the double phenyl structure restricts conformational flexibility, preventing optimal vertical alignment. Increased steric hindrance from the rigid hydrophobic segments forces the molecular axis to adopt a tilted or nearly planar orientation relative to the interface. As a result, hydrophobic groups are dispersed, and the contact area between hydrophilic groups and water is reduced. This tilted adsorption conformation leads to diminished exposure efficiency and solvation capacity, significantly compromising the com- pactness of the interfacial film.

M1: the tilt angle is approximately 80°, comparable to that of S1. The high proportion of single phenyl molecules dominates interfacial adsorption, and their conformational flexibility allows them to fill voids created by the rigid double phenyl components, maintaining a near-vertical orientation. The limited presence of rigid structures causes only minor perturbation to molecular alignment, enabling the system to preserve a small tilt angle. Consequently, the exposure efficiency of hydrophobic groups and the solvation degree of hydrophilic groups remain close to those observed in S1, resulting in the most densely packed interfacial film among all systems.

M2: the tilt angle is approximately 96°, the largest across all systems. At this medium blending ratio, the numbers of flexible (single phenyl) and rigid (double phenyl) molecules are similar, leading to competitive interactions at the interface. The conformational adaptability of the single phenyl units cannot fully accommodate the constraints imposed by the rigid double phenyl structures, resulting in molecular misalignment. This imbalance drives the molecular axis toward a highly tilted or flattened configuration, causing dispersion of hydrophobic domains and a substantial reduction in hydrophilic-water contact. The resulting adsorption conformation exhibits the lowest hydrophobic exposure efficiency, weakest solvation, and poorest interfacial film density.

M3: the tilt angle is approximately 91°, slightly lower than that of S2. The interfacial behavior is dominated by the rigid double phenyl molecules, which favor a tilted adsorption mode due to steric constraints. However, the presence of a small fraction of flexible single phenyl molecules partially mitigates the tilting effect by improving local packing, thereby reducing the average tilt angle compared to S2. This slightly optimized orientation enhances hydrophobic exposure and hydrophilic solvation relative to S2, yielding an interfacial film with marginally improved structural density.

There exists a distinct causal relationship between the tilt angle, the density distribution, and the surface tension. Specifically, as the tilt angle decreases, the peak of the density distribution becomes higher, the peak width becomes narrower, and the surface tension decreases. As depicted in Figure 4, when the tilt angle is smaller (vertical adsorption), the molecules are arranged more closely at the interface, leading to a high and narrow peak in the density distribution. In contrast, when the tilt angle is larger (tilted adsorption), the molecular arrangement is relatively loose, causing the peak of the density distribution to decrease and the peak width to increase. Figure 6 indicates that a smaller tilt angle facilitates the efficient upward aggregation of hydrophobic groups, thereby significantly reducing the surface tension. Conversely, a larger tilt angle results in the dispersion and exposure of hydrophobic groups at the interface, weakening their aggregation effect and consequently increasing the surface tension. Taking M1 as an example, it has the smallest tilt angle, corresponding to the highest peak and the narrowest peak width of the density distribution, as well as the lowest surface tension. On the contrary, M2 has the largest tilt angle, with the lowest peak and the widest peak width of the density distribution, and the highest surface tension. The microscopic mechanism of “decreasing tilt angle → enhanced concentration of density distribution → reduced surface tension” clearly reveals the regulatory law of molecular orientation on the interface performance.

#### 3.2.2 Solvent-Accessible Surface Area

The solvent-accessible surface area (SASA) serves as a fundamental parameter for quantifying the extent of contact between a molecule and a solvent. It can directly mirror the exposure status of hydrophilic and hydrophobic groups of surfactants at the gas/water interface and the solvation effect. By computing the regions on the molecular surface that are accessible to the solvent (e.g., water), it differentiates the hydrophilic surface area (such as the exposed area of sulfonate group) from the hydrophobic surface area (such as the exposed area of alkyl chains and benzene rings), thereby uncovering the correlation among molecular conformation, aggregation state, and interfacial properties. The hydrophilic surface area reflects the solvation capacity of hydrophilic groups and directly influences the stability of the hydration layer of the interfacial film. Meanwhile, the hydrophobic surface area indicates the degree of exposure of hydrophobic groups in the gas phase and determines the molecule’s ability to reduce surface tension. These two aspects work in harmony to jointly regulate the interfacial adsorption behavior and film stability of surfactants, and they represent the crucial foundation for analyzing the performance of compound systems.

In conjunction with the graph depicting the variation of the hydrophobic surface area over time (Figure 9) and supplemented by the hydrophilic surface area data, a more comprehensive analysis of the regulatory mechanism of the molecular structure and blending ratio on the solvation behavior of surfactants can be conducted.

**Figure 9:**
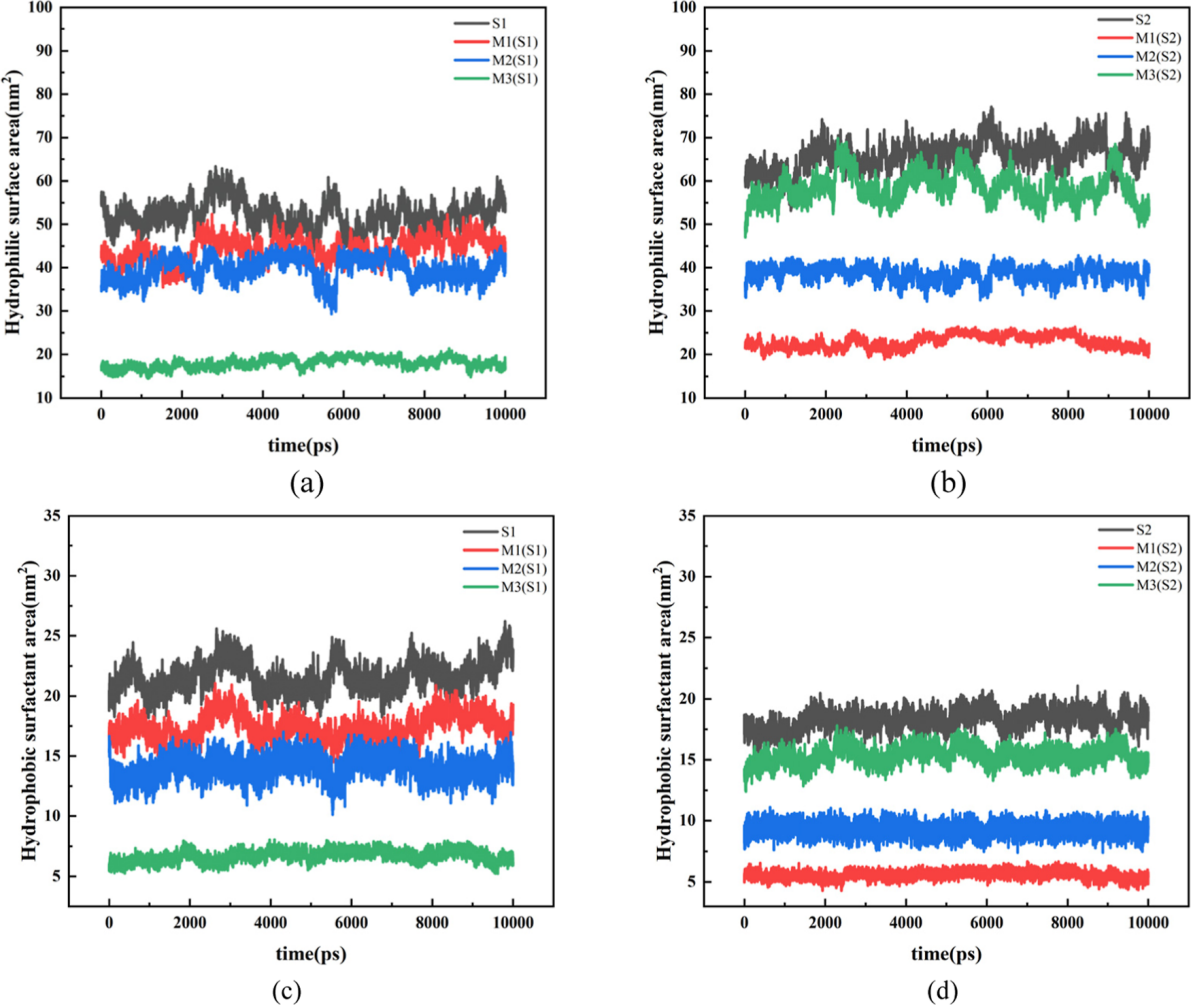
Hydrophilic and hydrophobic area curves of each system S1 and S2: (a,b) are the hydrophilic area curves of each systems S1 and S2; (c,d) are the hydrophobic area curves of each systems S1 and S2.

Within a single system, S1 (monophenyl, featuring a flexible structure) exhibits a relatively high hydrophilic surface area (approximately 55 nm2) with minor fluctuations. In contrast, the corresponding hydrophobic surface area (approximately 20 nm2) fluctuates more notably, ranging from approximately 18 to 25 nm^2^. The conformational freedom of the flexible alkyl chain gives rise to the dynamic alteration of the exposed area of the hydrophobic groups (benzene ring and alkyl chain) in the gas phase. However, the hydrophilic groups (sulfonate group) maintain stable contact with the aqueous phase through “vertical” adsorption, thereby achieving efficient separation of hydrophilic and hydrophobic surfaces and a high degree of solvation. S2 (diphenyl, characterized by a rigid structure) possesses a relatively high hydrophilic surface area (approximately 70 nm2), yet it experiences substantial fluctuations (ranging from approximately 60 to 80 nm^2^). Similarly, the corresponding hydrophobic surface area (approximately 18 nm^2^) also fluctuates significantly, ranging from approximately 15 to 22 nm^2^. The rigid double-benzene rings constrain the molecular conformation, resulting in the “disordered” exposure of the hydrophilic groups (partially obstructed by hydrophobic groups). Simultaneously, the exposure area of the hydrophobic groups in the gas phase fluctuates more extensively due to the tilted/lying adsorption of the molecule, leading to low separation efficiency of hydrophilic and hydrophobic surfaces and weak solvation stability.

In the mixed system, M1 exhibits a relatively high hydrophilic surface area (approximately 45 nm2), whereas the hydrophobic surface area (approximately 15 nm^2^) shows moderate fluctuations (ranging from approximately 12 to 18 nm^2^). A high proportion of flexible S1 dominates the interface, where alkyl chains “fill” the voids of rigid S2, thereby optimizing the exposure of hydrophilic groups (ensuring hydrophilic surface stability). Simultaneously, the rigidity of S2 “supports” the hydrophobic groups of S1, reducing their disordered exposure (mitigating hydrophobic surface fluctuations). The hydrophilic and hydrophobic surfaces operate synergistically to enhance solvation efficiency. For M2, the hydrophilic surface area decreases to approximately 35 nm^2^, and the hydrophobic surface area (approximately 10 nm^2^) experiences less fluctuation (ranging from approximately 8 to 12 nm^2^). At a medium proportion, the “conformational competition” between S1 and S2 results in an imbalance in hydrophilic surface exposure (with some sulfonate group being blocked). However, the hydrophobic surface fluctuation is reduced due to the “compact” molecular arrangement, and the synergy between the hydrophilic and hydrophobic surfaces weakens. In the case of M3, the hydrophilic surface area is the lowest at approximately 20 nm, and the hydrophobic surface area (approximately 7 nm^2^) has minimal fluctuations (ranging from approximately 5 to 8 nm^2^). A high proportion of rigid S2 dominates the interface, and the “dispersed” adsorption of molecules causes the hydrophilic groups to be largely blocked by hydrophobic benzene rings (resulting in minimal hydrophilic surface exposure). Meanwhile, the hydrophobic groups, due to their tight arrangement, have the lowest fluctuation. Nevertheless, the low exposure of the hydrophilic surface leads to a significant decrease in the solvation degree.

The adsorption morphology of styrene-based unsaturated cardanol sulfonate surfactants at the gas/water interface is intricately associated with the solvent - accessible surface area (SASA). A smaller tilt angle implies that the molecules are more prone to adsorb vertically. In this state, the hydrophobic groups aggregate upward with high efficiency, and the hydrophilic groups are fully solvated. This leads to a high peak and a narrow peak width in the density distribution, along with a reduction in surface tension, as exemplified by the M1 system (tilt angle 80°, surface tension 66.9 mN/m). The SASA data further validate that in the M1 system, which has a high proportion of monophenol, the hydrophilic surface area remains stable, and the hydrophobic surface area fluctuates moderately. The synergy between flexibility and rigidity optimizes solvation, rendering the interfacial film dense and stable. In contrast, in the M2 system, which has a medium proportion of monophenol, due to conformational competition, the hydrophilic surface area decreases, the fluctuation of the hydrophobic surface area is reduced, the hydrophilic–hydrophobic synergy weakens, and the interfacial performance deteriorates. Both scenarios indicate that the molecular orientation and the degree of solvation jointly regulate the interfacial performance. A high proportion of monophenol in the formulation can attain the optimal adsorption morphology and solvation, offering a theoretical foundation for performance optimization.

### 3.3 Intermolecular Forces

The aggregation behavior and performance disparities of molecules at the gas/water interface are fundamentally determined by the type, intensity, and spatial distribution of intermolecular forces. The radial distribution function (RDF) and hydrogen bond number statistics, serving as the core methods for analyzing the local structure and interactions between molecules, can quantitatively disclose the interaction patterns between key groups such as sulfonate group and water, benzene rings, and alkyl chains from the dimensions of distance distribution and quantity. In this research, the RDF curves of sulfonic acid sulfur atoms (S) and water molecule oxygen atoms (O) in different systems (S1, S2, M1–M3) were computed, and the microscopic mechanism of interfacial behavior was elucidated from the perspective of intermolecular forces by integrating the data of hydrogen bond numbers.

The sulfonate group (−SO_3_^–^) functions as the hydrophilic group of anionic surfactants, and its hydrogen-bonding interaction with water molecules is the key factor for maintaining the stability of the interfacial film. The radial distribution function (RDF) curve can reflect the distance characteristics and orderliness of hydrogen-bonding interactions, whereas the quantity of hydrogen bonds directly indicates the strength of the interaction. The combination of these two aspects can comprehensively disclose the regulatory law of hydrogen bonds on interfacial behavior. From the radial distribution function (RDF) curves presented in Figure 10, it is evident that O–O interactions exhibit significant variation across the different systems. In the single-phenyl system, the O–O RDF profile for S1 displays a sharp primary peak at 0.27 nm (*g*_r_ ≈ 5.4), with a narrow full width at half-maximum of approximately 0.02 nm, along with a distinct secondary coordination shell peak at 0.45 nm (*g*_r_ ≈ 2.5). These features indicate the formation of a strong and highly ordered hydrogen-bonding network between the sulfonate group and surrounding water molecules. The flexible alkyl chain, coupled with a single phenyl group, enables the sulfonic acid moieties to fully extend into the aqueous phase, facilitating close-range (0.27 nm), stable hydrogen bonding interactions. This results in a well-organized first hydration shell, while the residual weak interaction in the second coordination layer further stabilizes the overall hydration structure. This structural organization is consistent with the high-density distribution peak observed for S1 (≈300), its narrow adsorption peak width, and the correspondingly low surface tension (68.4 mN/m). In contrast, for S2—characterized by a rigid structure incorporating two phenyl groups—the main O–O RDF peak is reduced in intensity (*g*_r_ ≈ 4.5) and broadened, with the complete absence of the second coordination shell peak. The constrained molecular geometry promotes tilted adsorption at the interface, limiting the penetration of sulfonate group into the aqueous phase and increasing their average distance from water molecules to 0.28 nm, thereby weakening hydrogen bond strength. Furthermore, the structural rigidity induces disordered orientation of the sulfonate group, reducing the structural order of water molecules in the first coordination layer and disrupting any residual long-range interactions. This microstructural disorder correlates with a lower density distribution peak for S2 (≈220), a broader peak width (≈4 nm), and a slightly elevated surface tension (68.5 mN/m).

**Figure 10:**
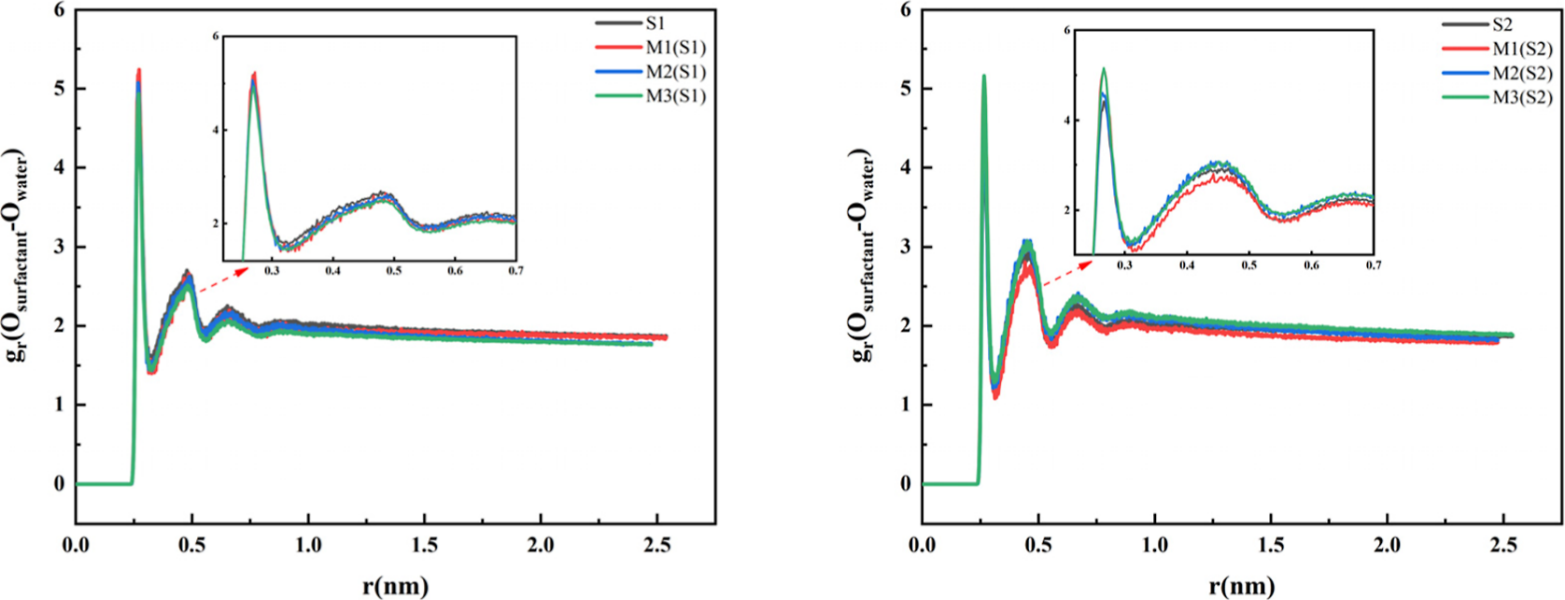
Radial distribution function curve graph.

In the mixed system, the M1, although the introduction of the biphenyl component causes a slight decrease in the main peak height (*g*(*r*) ≈ 4.8), the peak position remains unchanged, suggesting that the sulfonate groups can still effectively contact the aqueous phase and maintain strong hydration. This microstructure aligns with the low surface tension of M1, implying that blending leads to tighter molecular packing at the interface with fully exposed polar groups, thereby reducing surface tension more effectively. As the biphenyl ratio increases to M2, the main peak height further decreases (*g*(*r*) ≈ 4.5) and the peak width broadens, indicating intensified conformational competition where some sulfonate groups are shielded by hydrophobic moieties, leading to reduced hydration stability and a slight rebound in surface tension (67.6 mN/m). In the M3 system, the main peak height drops significantly (*g*(*r*) ≈ 3.8) with the maximum peak width, demonstrating that a high proportion of rigid biphenyl groups severely hinders the contact between sulfonate groups and water, resulting in extremely weak hydration and disordered interfacial arrangement. This corresponds to a substantial increase in surface tension to 68.1 mN/m, approaching the level of S2. Additionally, the main peak for the S2 system is closer to the origin (≈ 0.35 nm) with lower intensity, indicating that its molecular rigidity leads to weaker sulfonate hydration and inferior surface activity compared to S1. In conclusion, the RDF and surface tension data collectively demonstrate that blending at moderate ratios can optimize molecular conformation and enhance interfacial hydration, maximizing surface activity, whereas an excessive proportion of rigid components disrupts the hydration layer structure and diminishes surface activity.

The statistical analysis of hydrogen bond counts (Figure 11) further corroborates the conclusions drawn from the RDF data. A clear trend is observed in the number of hydrogen bonds across the systems: S1 ≈ S2 > M1 ≈ M2 > M3. The hydrogen bond counts for S1 and S2 are comparable and exhibit the highest values, indicating that the hydrophilic sulfonate group in both monophenyl and diphenyl sulfonates are capable of forming extensive hydrogen bonding interactions with water molecules. In the mixed systems, the hydrogen bond count gradually decreases with increasing proportion of diphenyl groups, yet M1—dominated by monophenyl moieties—retains a relatively high value. Specifically, S1 displays the highest main RDF peak and the largest hydrogen bond count, demonstrating that the hydrogen bonds between sulfonate group and water molecules are not only numerous but also spatially well-ordered. The flexible alkyl chain enables full exposure of the sulfonate group to the aqueous phase, facilitating the formation of a dense and stable hydrogen-bonding network. This structural feature supports high-density interfacial adsorption and contributes to low surface tension. In contrast, although the main RDF peak for S2 is significantly reduced and broadened, its hydrogen bond count decreases only slightly, suggesting that while the total number of hydrogen bonds remains largely preserved, their structural orderliness is markedly diminished. The rigidity imposed by the diphenyl structure leads to tilted and dispersed orientations of the sulfonate group, resulting in a disordered spatial distribution of hydrogen bonds. Despite maintaining a considerable quantity, the weakened interaction strength reduces interfacial packing efficiency and lowers adsorption density. For M1, both the main RDF peak height and hydrogen bond count remain relatively high, indicating that under monophenyl dominance, the hydrogen-bonding network retains strong structural order. In M3, however, the main RDF peak is the lowest and the peak width is the largest, accompanied by the smallest hydrogen bond count, evidence that the prevalence of rigid diphenyl groups shields the sulfonic acid functionalities, leading to simultaneous reductions in both hydrogen bond quantity and spatial order. Consequently, the stability of the interfacial hydration layer is severely compromised. A combined analysis of RDF profiles and hydrogen bond statistics reveals a fundamental regulatory mechanism governing interfacial properties: the “numb- er–orderliness” synergy of hydrogen bonds jointly determines interfacial behavior. S1 achieves the lowest surface tension due to its high hydrogen bond count and strong structural order, reflecting a highly stable hydration environment. Although S2 exhibits a similar hydrogen bond count, the loss of spatial order weakens overall interaction strength, resulting in a marginally higher surface tension. In mixed systems, a high proportion of monophenyl groups preserves both hydrogen bond number and orderliness, which—combined with the hydrophobic effects inferred from RDF—leads to optimal interfacial film density. These hydrogen bond data provide robust validation of the RDF-based interpretation, confirming that the strength and structural organization of intermolecular hydrogen bonds are key determinants in modulating interfacial adsorption and surface tension, thereby offering multidimensional insights for optimizing surfactant performance.

**Figure 11:**
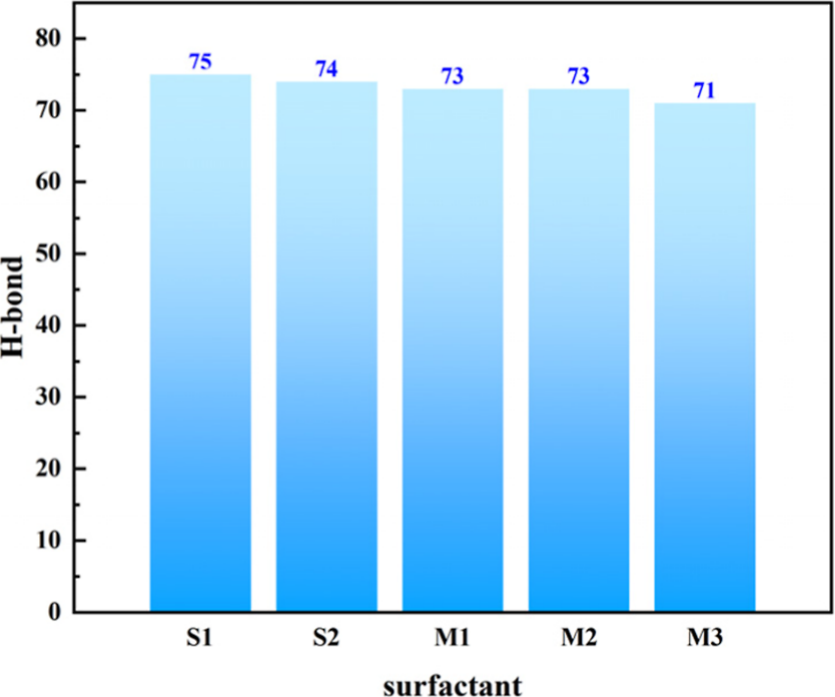
Hydrogen bond column charts of each system.

The combined analysis of radial distribution function (RDF) profiles and hydrogen bond counts reveals a fundamental regulatory mechanism by which hydrogen bonds govern interfacial performance: the “quantity–orderliness” synergy of hydrogen bonds jointly determines key interfacial properties. S1 exhibits the lowest surface tension due to its high number of hydrogen bonds and strong structural order, both of which contribute to a stable and well-organized interfacial hydration layer. Although S2 maintains a hydrogen bond count comparable to that of S1, the significant reduction in spatial order leads to weakened interaction strength, resulting in a slightly elevated surface tension. In the mixed systems, a high proportion of monophenyl groups—as exemplified by M1—preserves both the quantity and structural order of hydrogen bonds. This preservation, in conjunction with the hydrophobic effects inferred from RDF data, enables the formation of an optimally compact interfacial film. The hydrogen bond count data provide quantitative validation of the RDF-based interpretation, confirming that the strength and spatial organization of intermolecular hydrogen bonding are critical factors in modulating interfacial adsorption behavior and surface tension. These findings offer multidimensional insights for the rational design and performance optimization of surfactant molecules.

## 4 Conclusion

This study employs molecular dynamics simulations to elucidate the synergistic regulation mechanism of styrene-based unsaturated cardanol sulfonate surfactants at the air/water interface. By constructing models with varying blending ratios of monophenyl (flexible) and bis-phenyl (rigid) structures, we reveal how molecular architecture and composition govern interfacial assembly and macroscopic properties.

The key finding is that the interplay between molecular rigidity and blending ratio dictates the interfacial behavior. Specifically, a high proportion of monophenyl surfactants facilitates a “flexible-rigid synergy.” In this scenario, the flexible chains fill intermolecular voids while the rigid segments provide structural support, leading to dense vertical packing and optimized solvent accessibility. This synergistic arrangement significantly enhances the formation of a compact interfacial film, thereby reducing surface tension more effectively than single-component systems.

Furthermore, the study demonstrates that this synergistic effect is directly linked to the reinforcement of hydrogen bonding networks between the sulfonate headgroups and water molecules. The optimized molecular orientation in the blended system strengthens these interactions, which are crucial for stabilizing the interface.

In summary, this work provides a molecular-level blueprint for designing high-performance biosurfactants. By leveraging the complementary nature of flexible and rigid molecular structures, our findings offer a strategic approach to tune interfacial properties. This not only advances the theoretical understanding of surfactant self-assembly but also paves the way for developing more efficient, biobased surfactants for applications in green chemical engineering and industrial formulations.

## Supplementary Material



## Notes

Molecular dynamics simulation input files (including.itp, .top topology files and.pdb structure files for D-SUCS and M-SUCS surfactants), supplementary figures, and full experimental details which is archived in the compressed file Q1-S1 (ZIP)
